# A Peculiar Case of Functional Derangement of the Skin in a Dog, Manifested by Hemorrhages and Abnormal Secretions, Followed by Death from Acute Infection

**Published:** 1900-06

**Authors:** Cecil French

**Affiliations:** Washington, D. C.


### DEPARTMENT OF CANINE AND FELINE MEDICINE AND SURGERY.

### A PECULIAR CASE OF FUNCTIONAL DERANGEMENT OF THE SKIN IN A DOG, MANIFESTED BY HEMORRHAGES AND ABNORMAL SECRETIONS, FOLLOWED BY DEATH FROM ACUTE INFECTION.

By Cecil French, D.V.S.,
Washington, D. C.

Subject: Smooth-coated mongrel bitch, weighing about fifteen pounds, and aged about thirteen years at the time of her death, in the autumn of 1898.

This animal enjoyed good health until the year 1895, when, according to the owner's statement, symptoms of bloating, ravenous appetite, and unquenchable thirst appeared. Vomiting frequently occurred when she was permitted to indulge her appetite. The inordinate appetite for food and drink continued until three or four days before dissolution. Constant micturition of pale urine was also a prominent symptom.

In other respects nothing seemed amiss until the summer of 1898, when the owner noticed the sudden appearance of rather profuse cutaneous hemorrhages. The exuded blood dried on the coat in the form of little dark-brownish grits. When the animal was washed these grits dissolved and transmitted to the water the color and odor of blood. I may mention that the owner was averse to submitting the animal to any treatment, and I was only allowed the opportunity of observing the appearance of the blood coagula on one occasion just prior to washing. I secured some of the material and submitted it to an expert for spectroscopic analysis, who confirmed the opinion I held as to its character.

While washing the animal one day, some two months subsequent to the first appearance of the bleeding, the owner noticed that pressure on the skin caused the exudation of a secretion which was described to me as being of paste-like consistence and spiral worm-like form, about an inch in length and as thick as a darning needle (abnormal sebum?). The orifices from which the secretion could be forced were quite numerous, and the latter exhibited a regularity of shape which suggested a tubular origin. In fact, it closely resembled the form artists' oil-paint assumes on being squeezed out of the tubes in which it is kept. It sank to the bottom of the wash-tub without showing any tendency to dissolve. Scabs and crusts were forming during this period. When the same peeled off the hair came with them, and failed to grow again, so that latterly the animal was almost entirely denuded of hair.

About the beginning of December the manifestations of skin trouble ceased. She continued to grow feeble, but without showing any sign of emaciation, until during the last days of February she suddenly went all to pieces and died on the 4th of March. Toward the end the fecal discharges were very loose and fetid.

I was permitted to make an examination after death, and observed the following conditions present:

*Small* and *large gut* much inflamed and ulcerated. Several well-developed tapeworms present.

*Liver* rough on surface and peculiarly mottled, the mottling extending to the tissue within. Gall-bladder empty.

*Spleen* shrunken and mottled at each extremity like the liver.

*Kidneys* anæmic. Urine from the bladder contained albumin, but no sugar.

*Heart* greatly enlarged.

The microscope demonstrated the presence in the intestine, liver, and spleen of a microörganism of diplococcus form.

I submitted sections of the diseased organs as well as agar cultures from the same to Dr. Adami, of the McGill Pathological Laboratory, and received from him the following remarks:

"Going over your material I find it interesting so far as regards points on which I am at present working, more especially in connection with the liver in the case. This shows a very extreme condition of development of focal necrosis. I have never seen so extreme a condition in man, save in rare cases of eclampsia in connection with childbirth. The condition has been evidently of some days' duration, certainly not of months or years. One notes in parts irregular small areas in which the liver-tissue does not stain, but the cells are still recognizable by their outlines; here evidently the necrosis has been very recent. But cheek-by-jowl with these are other areas of about the same size, densely infiltrated with small round cells; this is a late stage in which there has been invasion of the necrotic areas by leucocytes; the organ further shows distinct congestion. The skin is normal, though the relative absence of hair follicles is rather striking. I make out in it no special indications of inflammatory change; if anything, it is atrophied rather than hypertrophied. The microörganism in every respect conforms to the cultural characteristics of the colon bacillus. It is not at all surprising that with enteritis you find the colon bacillus in various organs, more especially in the liver and spleen; whether that has been the cause of the condition seen in the liver I dare not say. It might be. In other animals it is unusual to get so extreme a condition. I have stained sections of the liver with carbol thionin, and can recognize none of the ordinary bacteria, though there are minute diplococcus forms which are possibly colon bacilli undergoing destruction. I may further add that the condition of the liver is, we may almost certainly say, infective, for it is in infectious diseases such as typhoid and diphtheria that we most frequently meet this condition. One thing is clear, that the condition is a recent one. There is no evidence of its being chronic, and as a consequence we may say that the enteritis and the lesions which I have described in connection with the liver have no clear relationship to the cutaneous hemorrhages and earlier disturbances, though of course an animal in such bad health might be predisposed to enteritis and allied conditons.''

From a consideration of the early symptoms in this case, it would appear that the animal became infected with tapeworm at the outset. The bloating and inordinate appetite would account for this. But can we attribute to the action of the worms the diabetes which seems to have been present? We know that tapeworm infestation will produce remote effects on the system, possibly through reflex nervous influence, or absorption of the excretory products of the worms or other bodies resulting from or accompanying disordered digestion. On more than one occasion I have observed eruptive disorders of the skin to disappear on eradication of tapeworm, which would seem to indicate that they depended on the presence of the latter in the intestinal canal. But in this case we have something more than a mere eruption, evidently a disordered glandular function. Some of the greatest exponents of the physiological and pathological workings of the nervous system might not deem it unreasonable to lay the blame of all this cutaneous disturbance to reflex impulses.

With regard to the cutaneous hemorrhages, I have since noticed the same condition only in much milder form in a case of fatal distemper (nervous form). The owner, a lady, drew my attention to it by showing me the little coagulated blood grits which she had combed out of the dog's hair every morning while he was in a prostrated condition. It would appear during the night, and on dissolving it in water emitted the odor of blood.

The enteric infection could, of course, be ascribed to the damage done by the worms to the wall of the bowel, and this is what probably killed the animal.

